# Efficacy and safety of moxibustion in the treatment of female stress urinary incontinence: A protocol for systematic review and meta-analysis

**DOI:** 10.1097/MD.0000000000028893

**Published:** 2022-02-18

**Authors:** Yueyu Zhang, Zhongyu Zhou, Dan Wei, Yang Jiao, Qiaochu Zhu, Yue Shi, Baoyi Peng, Yangpu Zhang, Aiqun Song

**Affiliations:** a Hubei University of Chinese Medicine, Wuhan, China; b Department of Acupuncture, Hubei Provincial Hospital of Chinese Medicine, Wuhan, China; c Hubei Province Academy of Traditional Chinese Medicine, Wuhan, China; d Hubei Provincial Hospital of Integrated Chinese and Western Medicine, Wuhan, China.

**Keywords:** female, moxibustion, protocol, stress urinary incontinence, systematic review

## Abstract

**Background:**

Stress urinary incontinence (SUI) is one of the common diseases in female urinary system diseases, and the incidence is increasing year by year. Moxibustion therapy, as a kind of acupuncture therapy, has been widely used in the clinical treatment of SUI, but its therapeutic effect and safety have not been scientifically and systematically evaluated. Therefore, the protocol of this systematic review we propose this time is to scientifically evaluate the effectiveness and safety of moxibustion in the treatment of female stress urinary incontinence (FSUI).

**Methods:**

The following 8 electronic databases will be searched from establishment to December 2021: PubMed, Web of Science, Cochrane Library, Embase, China National Knowledge Infrastructure, VIP Database, Wanfang Database, China Biology Medicine disc. All randomized controlled trials of moxibustion in the treatment of FSUI will be searched in the above electronic databases. Two reviewers will independently complete research selection, data extraction, and research quality evaluation. After screening the studies, the quality of the included studies will be evaluated according to the quality standards specified in the Cochrane Handbook for Systematic Reviews of Interventions (version 5.1.0). The primary outcome of included studies is the change from baseline in urine leakage measured by the 1-hour pad test. Secondary outcomes include: the short-form of the International Consultation on Incontinence Questionnaire, the mean 72-hour urinary incontinence episode frequency, self-assessment of the patient's treatment effect, severity of urinary incontinence, and adverse events. Two reviewers will independently conduct study selection, data extraction, risk of bias assessment, and study quality assessment. And the STATA 14.0 software will be implemented for data synthesis and meta-analysis.

**Results:**

The result of this meta-analysis will be submitted to peer-reviewed journals for publication, and a comprehensive review of current evidence will be conducted.

**Conclusions:**

The conclusion of this systematic review will provide evidence for judging whether moxibustion is a safer and more effective intervention for female stress urinary incontinence.

**Trial registration number:**

The protocol has been registered on INPLASY2021120052.

## 1 Introduction

Stress urinary incontinence (SUI) is defined by the International Continence Society as the involuntary leakage of urine caused by increased abdominal pressure when coughing or sneezing.^[^1^,^^2^^]^ Urodynamic examination shows that involuntary urine leakage will occur when the abdominal pressure increases without detrusor contraction. At the same time, female stress urinary incontinence (FSUI) is one of the common diseases that affect women's quality of life.^[2]^ Epidemiological studies have shown that there are large differences in the prevalence of SUI in different cases, which may be related to the definition of the disease, the measurement method, the characteristics of the research object, and the investigation method. A study conducted in the United States reports that approximately 50% of adult women are affected by SUI.^[3]^ According to the results of a survey based on urinary incontinence population in China, the prevalence of SUI in adult women is 14%. It is more prevalent than the rate of urgency urinary incontinence and mixed urinary incontinence.^[4]^ SUI has become a global problem for women. More than 40% of women worldwide suffer from urinary incontinence.^[5]^ Due to the fear of SUI symptoms and the lack of medical awareness of urinary incontinence, >80% of women do not receive any treatment.^[6]^ SUI has caused great stress on the psychology of female patients and led to a decline in the quality of life. At the same time, it also brings a lot of trouble and burden to the patients’ family life and social activities.^[^7^–^9^]^ Women with SUI are less likely to participate in social activities and physical exercise. Because of their lifestyle changes, they will increase their risk of lifestyle-related diseases, such as high blood pressure, diabetes, osteoporosis, and depression.^[^10^–^12^]^ In addition, SUI will bring a huge economic burden to female patients and the medical system.^[13]^ The United States spends >$12 billion annually on the treatment of SUI, and this amount continues to grow.^[13]^

Treatments for SUI include behavioral therapy, medication, physical therapy, and surgery. Duloxetine is the recommended drug for the treatment of SUI, but due to the large side effects and adverse reactions of the drug, the patient's tolerance is poor, so that the patients cannot take it for a long time.^[14]^ The American Urological Association recommends conservative treatment for mild and moderate SUI, which mainly includes pelvic floor muscle training (PFMT).^[15]^ Although PFMT has A-level evidence of efficacy according to evidence-based guidelines, it needs to be maintained for at least 3 months, which is difficult for most female patients.^[16]^ In China, due to the lack of professional rehabilitation trainers for guidance, it is often difficult for female patients with urinary incontinence to master PFMT, which makes it difficult to promote PFMT as a conservative therapy.^[^17^,^^18^^]^ Severe SUI patients often use surgical treatment as the main method.^[^6^,^^19^^]^ However, the choice of surgical treatment will also increase the potential risk of postoperative complications and the incidence of adverse events, such as postoperative urinary retention and urinary tract infections.^[20]^ Based on the problems of the above-mentioned various treatment methods, we now urgently need to find a safe and effective treatment method to better treat FSUI.

Moxibustion belongs to a branch of acupuncture therapy, which originated in ancient China and has been developed for thousands of years.^[21]^ Traditional Chinese medicine theory believes that moxibustion has the functions of warming the meridians and dredging collaterals, promoting blood circulation and removing blood stasis, dispelling cold, and relieving pain, etc. For SUI, moxibustion on the kidney meridian or bladder meridian can reinforce qi and promote the recovery of bladder function.^[22]^ Moxibustion and acupuncture are recommended by the National Institutes of Health as complementary and alternative therapies for the treatment of SUI.^[^23^,^^24^^]^ Moxibustion has been proven to be effective in treating urinary system diseases.^[25]^ Moxibustion is a nonpenetrating traditional Chinese medicine therapy, which is mainly used to treat and prevent diseases through the meridian and acupoints or the infrared heat stimulation and drug effects produced when moxa is burned in the diseased part. Because moxibustion has the advantages of simple operation, significant curative effect, high safety, low economic cost, and non-invasiveness, it has been accepted by more and more FSUI patients.^[^23^,^^26^^]^ Moreover, using moxibustion as an alternative therapy to treat FSUI can not only avoid the side effects of oral drugs and potential risks brought by surgery, but also reduce the physical and economic burden of FSUI patients.

Although many clinical research trials in China show that moxibustion is widely used in the treatment of FSUI,^[^27^–^30^]^ there is still a lack of systematic reviews and meta-analysis on the clinical efficacy of moxibustion in the treatment of FSUI. Therefore, the purpose of this study is to comprehensively evaluate the efficacy and safety of moxibustion in the treatment of FSUI. We will conduct a systematic review of the published literature, and through meta-analysis, we can draw reliable conclusions and provide new evidence support for the clinical treatment of FSUI.

## 2 Methods

### 2.1 Study registration

The protocol for this systematic review and meta-analysis has been registered in INPLASY (https://inplasy.com/) international prospective register of systematic reviews with the registration number INPLASY2021120052. This protocol is drafted under the guidance of the Preferred Reporting Items for Systematic Reviews and Meta-analysis Protocols (PRISMA-P) statement.^[31]^

### 2.2 Eligibility criteria

#### 2.2.1 Types of studies

We will include all randomized controlled trials (RCTs) of moxibustion in the treatment of FSUI, which will be reported in English or Chinese, regardless of region and publication status. We will exclude non-RCTs, animal experimental studies, retrospective studies, case reports, conference articles, expert experience, and reviews.

#### 2.2.2 Types of participants

Female patients who are clearly diagnosed as SUI, regardless of age, race, country, or disease course, can be included in the study as long as they meet the diagnostic criteria of the International Urinary Incontinence Association.

#### 2.2.3 Type of interventions

The treatment group will use any kind of moxibustion, including moxa cone moxibustion, moxa stick moxibustion, warm needle acupuncture, and other moxibustion methods (e.g., thermal moxibustion, thunder-fire moxibustion, Du moxibustion, etc). Moxibustion as the main treatment method combined with other conservative treatments (e.g., acupuncture, oral medication, PFMT, biofeedback, etc) will also be included. There are no restrictions on moxibustion methods, acupoint selection, treatment time, moxibustion materials, and treatment course.

#### 2.2.4 Types of comparisons

The control group will use conventional standard treatment, such as sham moxibustion, placebo, drug treatment, or blank control.

#### 2.2.5 Outcomes

##### 2.2.5.1 Primary outcomes

The primary outcome of included studies is the change from baseline in urine leakage measured by the 1-hour pad test.

##### 2.2.5.2 Secondary outcomes

Secondary outcomes include the short-form of the International Consultation on Incontinence Questionnaire, the mean 72-hour urinary incontinence episode frequency, self-assessment of the patient's treatment effect, severity of urinary incontinence, and adverse events.

### 2.3 Exclusion criteria

(1) Types of study subjects and interventions that do not meet inclusion criteria will be excluded (e.g., other types of urinary incontinence).
(2) non-RCTs reviews, case reports, observational studies, animal studies, expert experience, and conference articles on SUI will be excluded.
(3) Articles with missing data and duplicate publications will be excluded.

### 2.4 Search methods for identification of studies

#### 2.4.1 Electronic searches

We will search the following databases from their inception to December 2021: PubMed, Web of Science, Cochrane Library, Embase, China National Knowledge Infrastructure, VIP Database, Wanfang Database, China Biology Medicine disc. In addition, we will also search trial registration platforms, including International Clinical Trials Registry Platform (https://clinicaltrials.gov/) and Chinese Clinical Trial Registry Centre (http://www.chictr.org.cn/), for ongoing or unpublished trials. We will search all of the above databases and websites using a combination of Medical Subject Headings and free words terms to ensure that all relevant articles are retrieved. The languages of included studies will be restricted in English and Chinese. The main search terms will include: “stress incontinence,” “moxibustion,” “randomized controlled trial.” The search strategy of PubMed will be shown in Table 1. Similar retrieval strategies will be applied to the other databases mentioned above.

**Table 1:** The search strategy used in the PubMed database.

Search number	Query
#1	Urinary Incontinence, Stress [MeSH Terms]
#2	((Urinary Stress Incontinence [Title/Abstract]) OR (Incontinence, Urinary Stress [Title/Abstract])) OR (Stress Incontinence, Urinary [Title/Abstract])
#3	#1 OR #2
#4	Moxibustion [MeSH Terms]
#5	(((((((((moxabustion [Title/Abstract]) OR (moxa cone moxibustion [Title/Abstract])) OR (moxa stick moxibustion [Title/Abstract])) OR (warm needle acupuncture [Title/Abstract])) OR (thermal box moxibustion [Title/Abstract])) OR (needle warming therapy [Title/Abstract])) OR (direct moxibustion [Title/Abstract])) OR (indirect moxibustion [Title/Abstract])) OR (thermal moxibustion [Title/Abstract])) OR (thunder fire moxibustion [Title/Abstract])
#6	#4 OR #5
#7	randomized controlled trial [Publication Type]
#8	(((Randomized Controlled Trials as Topic [MeSH Terms]) OR (Clinical Trials, Randomized [Title/Abstract])) OR (Trials, Randomized Clinical [Title/Abstract])) OR (Controlled Clinical Trials, Randomized [Title/Abstract])
#9	#7 OR #8
#10	#3 AND #6 AND #9

(From establishment to December 2021).

#### 2.4.2 Searching other resources

We will also search for relevant systematic reviews of moxibustion for FSUI and reference lists of eligible studies to ensure that all studies that meet the inclusion criteria are included.

### 2.5 Data collection and analysis

#### 2.5.1 Selection of studies

**Figure 1:**
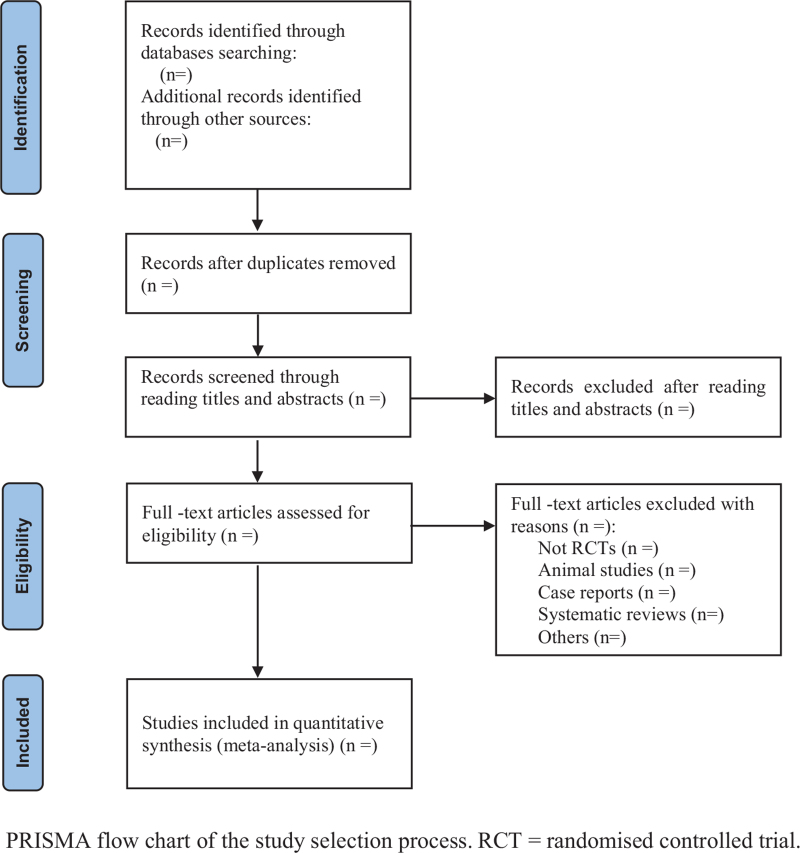
PRISMA flow chart of the study selection process. PRISMA = Preferred Reporting Items for Systematic Reviews and Meta-analysis, RCT = randomized controlled trial.

All literature retrieved from the electronic database will be imported into NoteExpress V.3.2.0 software (Beijing Aiqihai Software Company. Beijing Aiqi Haole Technology Co., Ltd) for classification management. First, duplicate published literature will be excluded. Then, 2 reviewers (QZ and BP) will independently read the titles and abstracts of the obtained articles, and screen the eligible articles according to the inclusion criteria. Finally, 2 reviewers will read the full text to further determine whether the remaining articles will be eligible for inclusion. Two reviewers will cross-check literature screening results. If 2 reviewers disagree during the literature search and literature screening process, a third reviewer (YZ) will be consulted and discussed with and a consensus will be reached. The detailed process of study selection will be shown according to the PRISMA flow chart (Fig. 1).

#### 2.5.2 Data extraction and management

Two reviewers (YS and YZ) will independently extract the following data from included studies:

(1) Basic information: first author, title, journal, publication year, and country.
(2) Participant's characteristics: age, race, country, disease course, and sample size.
(3) Methodological characteristics: interventions, comparisons, risk of bias assessment, method of randomization, and blinding method.
(4) Outcomes, follow-up, and adverse events.

If there is a disagreement during the data extraction process, the 2 reviewers will discuss and reach an agreement. If no consensus is reached, the third reviewer (ZZ) will be consulted and the disagreement will be resolved.

#### 2.5.3 Assessment of risk of bias

The risk of bias of included studies will be assessed using the Cochrane Collaboration Risk of Bias Tool.^[32]^ The Risk of bias includes the following 7 aspects: random sequence generation; allocation concealment; blinding participants and personnel; blinding of outcome assessment; incomplete outcome data; selective reporting; other bias. The risk assessment will be divided into 3 levels: low risk, high risk, and unclear risk. The evaluation of the above content will be completed by 2 reviewers (DW and YJ) independently, and any discrepancies will be resolved through discussion or negotiation with the third reviewer (AS).

#### 2.5.4 Measurement of treatment effect

Statistical management software (STATA 14.0; Texas, USA) will be used for data processing and meta-analysis. For dichotomous data, relative risk with 95% confidence interval (CI) will be used as the measure. For continuous data, either mean difference (MD) or standard mean difference with 95% CI will be selected for analysis.

#### 2.5.5 Dealing with missing data

If data are missing from included studies, we will contact corresponding authors by phone or email to obtain missing data. If missing data cannot be obtained, the study will eventually be excluded and our analysis will be based on available data.

#### 2.5.6 Assessment of heterogeneity

Heterogeneity of included studies will be assessed using the chi-square test (test level *α* = 0.1) and *I* ^2^ statistic.^[33]^ The fixed effect model or random effects model will be chosen depending on the *I* ^*2*^ statistic. When *I* ^2^ < 50%, there is less heterogeneity between studies and we will use a fixed effects model. If *I* ^2^ ≥ 50%, indicating significant heterogeneity between studies, we will choose a random effects model. When there is significant heterogeneity in the study results, we will choose subgroup analysis or sensitivity analysis to seek possible sources from clinical and methodological perspectives.

#### 2.5.7 Assessment of reporting biases

We will use funnel plots to assess publication bias when there are ≥10 RCTs eligible for inclusion.^[34]^ The funnel plot asymmetry will be assessed by Egger test.

#### 2.5.8 Data synthesis

Meta-analyses will use STATA 14.0 statistical software for data synthesis. We will use relative risk with 95% CI for dichotomous variables. For continuous variables, we will use MD or standard mean difference with 95% CI. If there is no significant heterogeneity, we will use a fixed-effects model for data synthesis; otherwise, we will use a random effects model for data synthesis. Furthermore, if heterogeneity is considered significant, sensitivity or subgroup analyses will be generated to distinguish its origin. In cases where the data are insufficient for quantitative analysis, the review will only represent and summarize the evidence.

#### 2.5.9 Subgroup analysis

If there is significant heterogeneity in the included studies, we will conduct subgroup analysis according to the different types of moxibustion, the severity of SUI, the difference in moxibustion acupoint selection, and the course of treatment.

#### 2.5.10 Sensitivity analysis

If the inclusion study is still heterogeneous after subgroup analysis, we will conduct a sensitivity analysis based on methodological quality, sample size, and the impact of missing data to assess the source of heterogeneity and check the stability of the results. In addition, after excluding studies of low methodological quality, the meta-analysis will be re-performed.

#### 2.5.11 Evidence quality evaluation

Grades of Recommendations Assessment Development and Evaluation (GRADE) V.3.6 software will be used to evaluate the quality of evidence.^[35]^ According to the GRADE guidelines, the quality of evidence will be divided into 4 levels (very low, low, moderate, and high). Five factors will reduce the level of evidence quality (study limitation, inconsistency, indirectness, publication bias, and imprecision).

#### 2.5.12 Ethics and dissemination

Ethical approval is not necessary as this protocol is for system review only and does not involve private data. The findings of this study will be disseminated a peer-review publication or presented at a relevant conference.

## 3 Discussion

Urinary incontinence in women is a clinically more common urological disorder than any other chronic disease such as hypertension, depression, or diabetes.^[^36^,^^37^^]^ Among the various forms of urinary incontinence, SUI is the most common. The treatment of SUI is one of the greatest challenges facing women's health and quality of life. Surgery can be used when conventional treatments for FSUI do not work, but patients are often reluctant to undergo surgery, and surgery has potential risks and adverse effects. Patients who are reluctant to undergo surgery begin to seek help from complementary alternative therapies such as acupuncture and moxibustion.^[38]^ Conservative and non-invasive treatments have generally been shown to be effective in improving urinary incontinence symptoms.^[23]^ Compared with other conservative treatments, moxibustion is a non-penetrating treatment that does not cause any damage to the body, so patients have high compliance and good tolerance. Moxibustion has the advantages of non-invasiveness, high safety, low economic cost and remarkable curative effect, and is widely used in clinical treatment of FSUI.^[^27^–^29^]^ It is generally believed that moxibustion mainly exerts therapeutic effect through the thermal stimulation of the human body by thermal infrared rays and the pharmacological effects of moxa stick burning. Traditional Chinese medicine theory believes that moxibustion mainly treats FSUI by warming meridians and dredging collaterals, reconciling qi and blood, and balancing yin and yang. However, there is a lack of systematic review and meta-analysis of the efficacy and safety of moxibustion in the treatment of FSUI. Therefore, we will conduct a high-quality systematic review and meta-analysis to evaluate the efficacy and safety of moxibustion in the treatment of FSUI.

Moreover, this is the first protocol to comprehensively evaluate the clinical efficacy and safety of moxibustion in the treatment of FSUI. We hope that the results of this review will provide clinicians with more robust evidence-based evidence for the management of FSUI. Nevertheless, this review also has some limitations. First, the inclusion of literature included only Chinese and English medical databases. Therefore, we may miss some relevant literature published in other languages. Second, the quality of the included studies may not be of high quality, which will lead to significant heterogeneity.

## Author contributions

**Conceptualization:** Yueyu Zhang, Aiqun Song.

**Data curation:** Zhongyu Zhou, Yangpu Zhang, Aiqun Song.

**Formal analysis:** Dan Wei, Yang Jiao.

**Investigation:** Qiaochu Zhu, Yue Shi, Baoyi Peng.

**Methodology:** Yueyu Zhang, Zhongyu Zhou, Yangpu Zhang, Aiqun Song.

**Software:** Dan Wei, Yang Jiao.

**Supervision:** Yueyu Zhang.

**Writing – original draft:** Yueyu Zhang.

**Writing – review & editing:** Yueyu Zhang, Aiqun Song.
